# Supplementary material for: Vitamin D Modified DSS-Induced Colitis in Mice via STING Signaling Pathway
Source: Biology (Basel). 2025 Jun 18;14(6):715. doi: 10.3390/biology14060715 (PMC12190092; doi:10.3390/biology14060715)
Supplement: Supplementary file 1 [file biology-14-00715-s001.zip › Supplementary Materials S1: 16s-report of gut microbiota/Groups/diff_analysis/UnivarTestCAZY/figure/boxplot/top20.pdf]

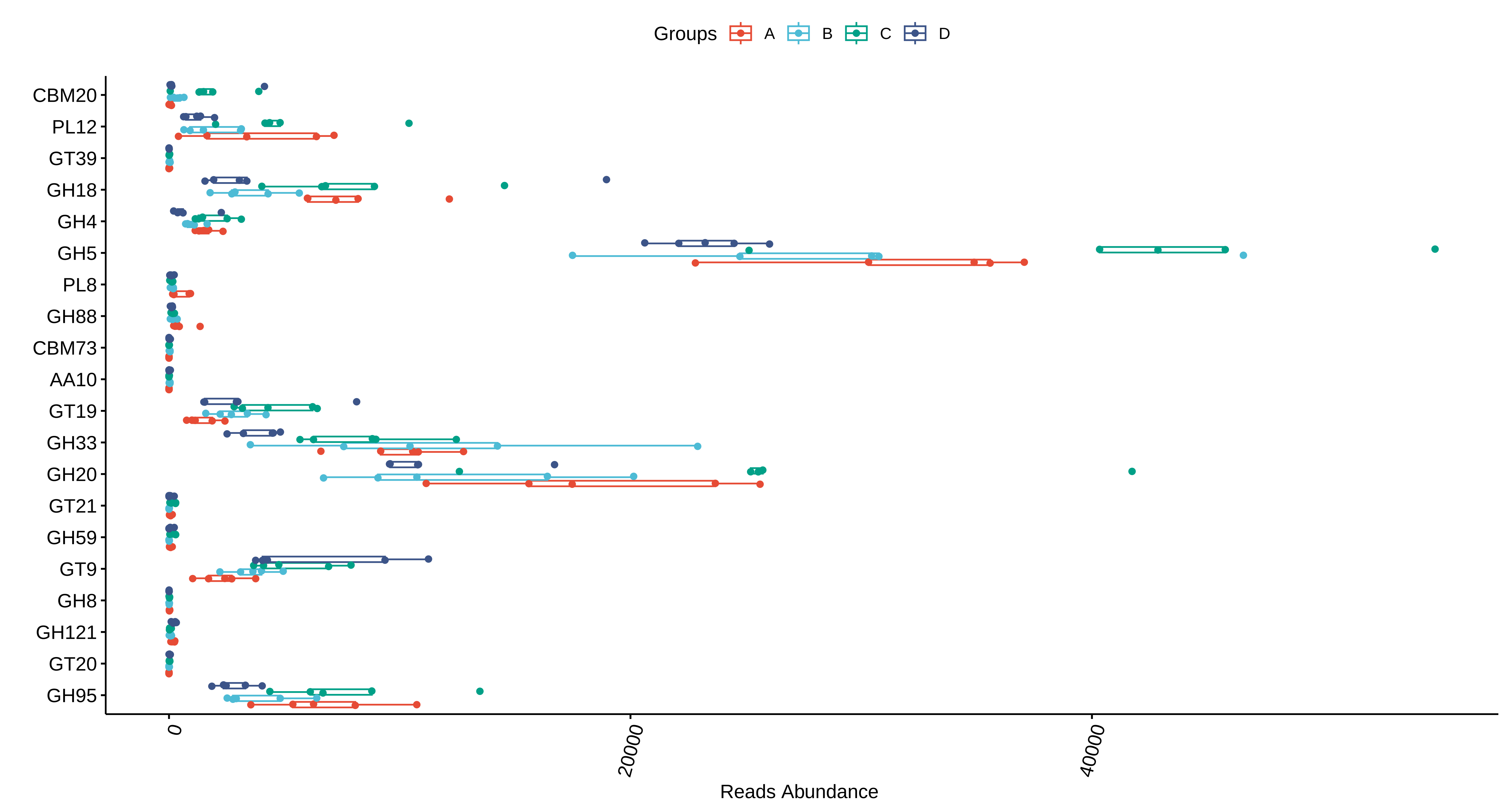
Groups
A
B
C
D
CBM20
PL12
GT39
GH18
GH4
GH5
PL8
GH88
CBM73
AA10
GT19
GH33
GH20
GT21
GH59
GT9
GH8
GH121
GT20
GH95
0
20000
40000
Reads Abundance
